# Chemoradiation with Cisplatin vs. Carboplatin for Squamous Cell Carcinoma of the Head and Neck (SCCHN)

**DOI:** 10.3390/cancers15133278

**Published:** 2023-06-21

**Authors:** Dirk Rades, Inga Zwaan, Tamer Soror, Christian Idel, Ralph Pries, Karl L. Bruchhage, Samer G. Hakim, Nathan Y. Yu

**Affiliations:** 1 Department of Radiation Oncology, University of Lubeck, 23562 Lubeck, Germany; inga.zwaan@uksh.de (I.Z.); tamer.soror@uksh.de (T.S.); 2 Department of Oto-Rhino-Laryngology & Head and Neck Surgery, University of Lubeck, 23562 Lubeck, Germany; christian.idel@uksh.de (C.I.); ralph.pries@uksh.de (R.P.); karl-ludwig.bruchhage@uksh.de (K.L.B.); 3 Department of Oral and Maxillofacial Surgery, University of Lubeck, 23562 Lubeck, Germany; samer.hakim@uni-luebeck.de; 4 Department of Oral and Maxillofacial Surgery, MSH Medical School Hamburg, Schwerin Campus, 19055 Schwerin, Germany; 5 Department of Radiation Oncology, Mayo Clinic, Phoenix, AZ 85054, USA; yu.nathan@mayo.edu

**Keywords:** head-and-neck cancer, chemoradiation, cisplatin, carboplatin, loco-regional control, metastases-free survival, overall survival, toxicity

## Abstract

**Simple Summary:**

Many head-and-neck cancer patients assigned to chemoradiation cannot receive concurrent cisplatin due to impaired renal function. Carboplatin may be an alternative option. We compared the chemoradiation outcomes of 131 patients who received cisplatin vs. 45 patients who were unfit for cisplatin and received carboplatin. Both groups were compared for loco-regional control, metastases-free survival, overall survival, toxicities, and the completion of chemotherapy. Patients receiving carboplatin were significantly older and had more G3 tumors; the other characteristics were balanced. No significant differences were found regarding loco-regional control, metastases-free survival, overall survival, and toxicities. Non-significantly more patients assigned to carboplatin completed their chemotherapy as planned. Given the limitations of this study, carboplatin appears a reasonable option for patients who require chemoradiation but cannot receive cisplatin.

**Abstract:**

Cisplatin is the standard for the chemoradiation of squamous cell carcinoma of the head and neck (HNSCC). Many patients cannot receive cisplatin due to impaired renal function. This study investigated carboplatin as an alternative option. In total, 131 patients assigned to two courses of cisplatin (20 mg/m^2^/d1-–5 or 25 mg/m^2^/d1–4) were matched to 45 patients not suitable for cisplatin and receiving carboplatin (AUC 1.0/d1–5 or AUC 1.5/d1–4). The endpoints included loco-regional control (LRC), metastases-free survival (MFS), overall survival (OS), toxicities, and the completion of chemotherapy. The patients in the carboplatin group were significantly older and had more G3 tumors. Otherwise, the baseline characteristics were balanced. The LRC rates at 2 and 3 years were 77% and 76% in the cisplatin group vs. 69% and 65% in the carboplatin group (*p* = 0.21). The MFS rates were 83% and 78% vs. 78% and 74% (*p* = 0.34) and the OS rates 83% and 79% vs. 83% and 75% (*p* = 0.64), respectively. The outcomes were not significantly different in the subgroups receiving definitive or adjuvant chemoradiation. No significant differences were found regarding toxicities. Non-significantly more patients in the carboplatin group completed their chemotherapy (78% vs. 66%, *p* = 0.15). Carboplatin was associated with similar outcomes and toxicities as cisplatin, although these patients had worse renal function, more aggressive tumors, and were older. Given the limitations of this study, carboplatin appears an option for patients not suitable for cisplatin.

## 1. Introduction

Many patients with locally advanced squamous cell carcinoma of the head and neck (HNSCC) receive concurrent chemoradiation with cisplatin, either as a definitive treatment or, in case of risk factors, following surgery [1,2,3]. However, many patients cannot receive cisplatin, mainly due to pre-treatment decreased renal function. Therefore, alternative systemic agents or combinations have been investigated, including carboplatin alone, carboplatin plus 5-fluorouracil (5-FU), carboplatin plus paclitaxel, mitomycin C plus 5-FU, or cetuximab. Randomized trials have demonstrated that chemoradiation with carboplatin plus 5-FU was superior to radiotherapy alone in patients with head-and-neck cancer [4,5,6,7,8,9,10]. In addition, a randomized trial from Germany found that the addition of mitomycin C plus 5-FU to hyper-fractionated accelerated radiotherapy resulted in significantly better loco-regional control (LRC) and overall survival (OS) [11,12].

However, several studies that included combinations with 5-FU found that these regimens were associated with significant acute toxicities [4,5,6,7,11,13,14]. For example, in the study of Hanemaaijer et al. that compared concurrent treatment with carboplatin plus 5-FU to cisplatin, more patients in the carboplatin plus 5-FU group discontinued their chemotherapy due to chemotherapy-related toxicities [13]. In another study, discontinuation of chemotherapy showed a trend towards worse OS in patients with oropharynx cancer [15]. Moreover, chemotherapy-related toxicity may lead to an interruption of the radiation treatment, which was shown to impair the patients’ prognoses [16,17]. Therefore, concurrent systemic therapies without 5-FU may be preferable for head-and-neck cancer patients unable to receive cisplatin. Such regimens may include cetuximab or carboplatin with or without paclitaxel [3,18,19,20].

A few studies compared radiotherapy plus concurrent cetuximab to concurrent chemoradiation with carboplatin-based regimens and found that the regimens including carboplatin resulted in better LRC and OS [21,22,23,24,25]. One of these studies differentiated between carboplatin-based combinations with 5-FU or paclitaxel and carboplatin as a single agent [23]. Carboplatin alone was not inferior to carboplatin-based combinations with respect to LRC, metastases-free survival (MFS), and OS [23]. Promising results regarding chemoradiation with carboplatin alone were also reported by other authors [26,27,28,29]. Several studies compared chemoradiation with concurrent carboplatin alone to concurrent cisplatin [30,31,32,33,34,35,36,37,38,39,40,41].

These studies produced conflicting results regarding treatment outcomes, suggesting the superiority of cisplatin (four studies), superiority of carboplatin (one study), or similar efficacy of both agents (six studies). One study found cisplatin to be superior for stage III but not for stage I or II tumors [39]. Moreover, these studies used radiotherapy techniques such as Cobalt60, 3D-conformal radiotherapy, and/or intensity-modulated radiotherapy but not volumetric modulated arc therapy (VMAT). This type of modern high-precision radiotherapy can be considered the new standard technique for treatment of SCCHN. Thus, additional comparative studies are required to better define the potential role of carboplatin alone for the chemoradiation of head-and-neck cancer. The present study compared two courses of concurrent carboplatin (AUC 1.0–1.5 on 4–5 days) and two courses of cisplatin (20–25 mg/m^2^ on 4–5 days, cumulative dose = 200 mg/m^2^). Since this cisplatin regimen was found to be similarly effective but significantly less toxic when compared to three courses of 100 mg/m^2^, two courses of fractionated cisplatin have become the standard regimen for SCCHN in our university hospital and selected as the reference treatment [42]. Our study is the first one that directly compared carboplatin and cisplatin for the chemoradiation of SCCHN in patients treated with VMAT.

## 2. Materials and Methods

A total of 176 patients treated with chemoradiation for SCCHN between 2012 and 2022 were included in this retrospective study, which was approved by the Ethics Committee of the University of Lübeck, Germany (file no. 21-034). Of the entire cohort, 131 patients were scheduled for concurrent chemoradiation including two courses of cisplatin, consisting of 20 mg/m^2^/d1–5 or 25 mg/m^2^/d1–4. In total, 45 patients could not receive cisplatin, mainly due to a decreased renal function, and were treated with two courses of concurrent carboplatin (AUC 1.0 on days 1–5 or AUC 1.5 on days 1–4) instead. Planned doses of external beam radiotherapy (EBRT), which was administered as VMAT, were 60–70 Gy with doses per fraction of 2 Gy, given on 5 consecutive days per week. The total radiation doses depended on the extent of resection and the presence of an extracapsular spread of lymph node metastases (ECS). The doses were 60 Gy after microscopically complete resection, 66 Gy after microscopically incomplete resection or ECS, and 70 Gy for definitive treatment. The median doses of EBRT were 66 Gy in the entire cohort and both treatment groups. Ten patients in the cisplatin group and one patient in the carboplatin group received a brachytherapy boost of 7.5–16 Gy with 3–5 fractions of 2.5–4 Gy.

Treatment groups were compared with respect to pre-treatment patient and tumor (=baseline) characteristics, outcomes in terms of LRC, MFS, and OS, completion of the planned chemotherapy, and for toxicities in terms of oral mucositis, dermatitis, xerostomia, cervical lymph edema, nausea, and hematotoxicity. The baseline characteristics included age (≤62 vs. ≥63 years, median age = 62 years), gender (female vs. male), Karnofsky performance score (KPS ≤80 vs. 90–100), the tumor site (oropharynx/oral cavity vs. hypopharynx/larynx vs. both), primary tumor stage (T1–2 vs. T3 vs. T4), nodal stage (N0-2a vs. N2b-3), histologic grade (G1–2 vs. G3), human papilloma virus (HPV) status (negative vs. positive), upfront surgery (no vs. yes), history of smoking prior to chemoradiation (no vs. yes), smoking during chemoradiation (no vs. yes), and the pre-treatment hemoglobin level (<12 vs. ≥12 g/dL). The distributions of these characteristics in both treatment groups are given in Table 1. In addition to the type of chemotherapy and completion of chemotherapy as planned, these characteristics were evaluated for associations with LRC, MFS, and OS.

Moreover, subgroup analyses comparing carboplatin and cisplatin with respect to LRC, MFS, and OS were performed for patients receiving definitive chemoradiation and for patients treated with adjuvant chemoradiation following surgery.

Statistical analysis regarding the comparison of the treatment groups with respect to distributions of baseline characteristics, completion of chemotherapy, and toxicities were performed with the Chi-square test or, in the case of fewer than 5 patients in one or more cells, with Fisher’s exact test. LRC, MFS, and OS were referenced from the last day of radiotherapy and the corresponding rates were calculated with the Kaplan–Meier method and the log-rank test (univariable analyses). The characteristics that were significantly associated with outcomes (*p* < 0.05) were included in a multivariable analysis (Cox proportional hazards model). For these analyses, the software BlueSky Statistics 10 GA was used (BlueSky Statistics LLC, Chicago, IL, USA).

## 3. Results

In the entire cohort, median periods of follow-up were 30 months (range: 0–110 months) in the cisplatin group and 24 months (range: 0–71 months) in the carboplatin group. The comparison of both treatment groups with respect to the patient and tumor characteristics revealed that significantly more patients in the carboplatin group were ≥63 years of age (64% vs. 41%, *p* = 0.007) and that more patients had poorly differentiated (G3) tumors (61% vs. 40%, *p* = 0.017). Otherwise, the patient and tumor characteristics were balanced between the groups (Table 1). In the carboplatin group, non-significantly more patients completed their chemotherapy as planned (78% vs. 66%, *p* = 0.15).

**Figure 1:**
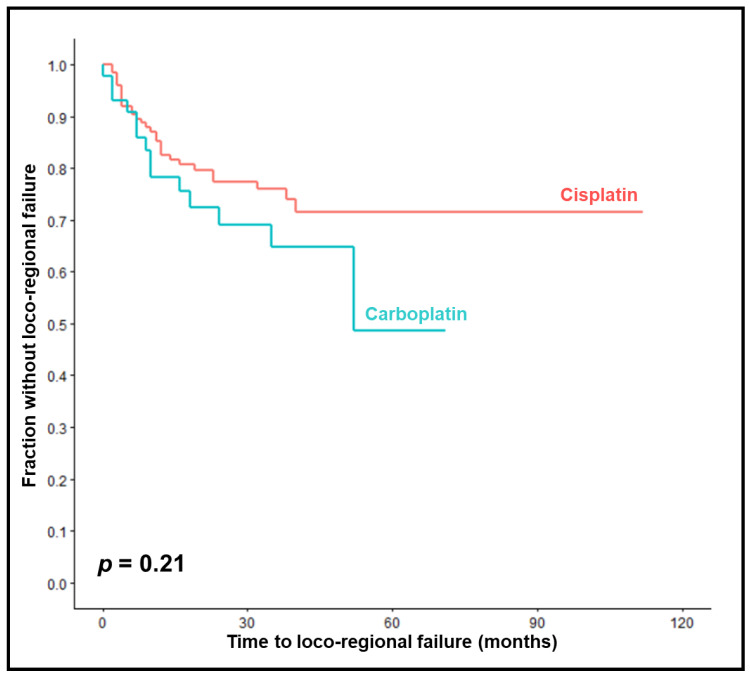
Comparison of chemoradiation with carboplatin vs. cisplatin with respect to loco-regional control (Kaplan–Meier curves). The *p*-value was calculated using the log-rank test.

LRC rates at 1, 2, and 3 years were 78%, 69%, and 65% in the carboplatin group compared to 83%, 77%, and 76% in the cisplatin group (*p* = 0.21, Figure 1). On univariable analyses, an improved LRC was significantly associated with KPS 90–100 (*p* = 0.049), a less advanced primary tumor stage (*p* < 0.001), a less advanced nodal stage (*p* = 0.004), upfront surgery (*p* < 0.001), and HPV positivity (*p* = 0.001) (Table 2). A trend was found for the completion of chemotherapy (*p* = 0.078). In the multivariable analysis of LRC, a less advanced primary tumor stage (*p* = 0.031) and HPV positivity (*p* = 0.047) were significant (Table 3).

**Figure 2:**
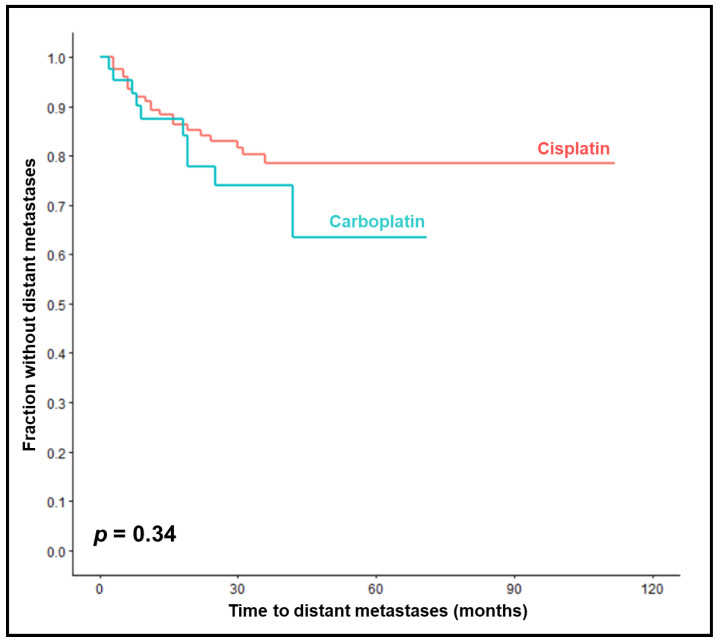
Comparison of chemoradiation with carboplatin vs. cisplatin with respect to metastases-free survival (Kaplan–Meier curves). The *p*-value was calculated using the log-rank test.

The 1-year, 2-year, and 3-year MFS rates were 87%, 78%, and 74% in the carboplatin group compared to 89%, 83%, and 78% in the cisplatin group (*p* = 0.34, Figure 2). On univariable analyses, improved MFS was significantly associated with KPS 90–100 (*p* = 0.031), a less advanced nodal stage (*p* = 0.013), and HPV positivity (*p* = 0.008) (Table 4). In the multivariable analysis of MFS, a less advanced nodal stage (*p* = 0.024) and HPV positivity (*p* = 0.030) were significant (Table 5).

**Figure 3:**
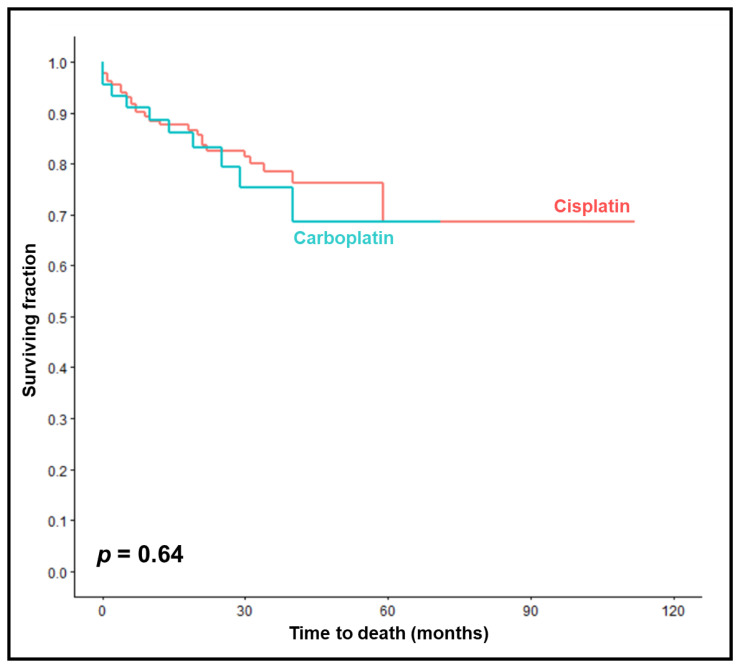
Comparison of chemoradiation with carboplatin vs. cisplatin with respect to overall survival (Kaplan–Meier curves). The *p*-value was calculated using the log-rank test.

OS rates at 1, 2, and 3 years were 89%, 83%, and 75% in the carboplatin group compared to 88%, 83%, and 79% in the cisplatin group (*p* = 0.64, Figure 3). On univariable analyses, improved OS was significantly associated with KPS 90–100 (*p* = 0.003), HPV positivity (*p* = 0.021), and not smoking during chemoradiation (*p* = 0.009) (Table 6). Trends were found for age ≤62 years (*p* = 0.093), pre-treatment hemoglobin levels ≥12 g/dL (*p* = 0.093), and the completion of chemotherapy (*p* = 0.060). In the multivariable analysis of OS, no characteristic achieved significance (Table 7).

Moreover, no significant differences were found between carboplatin and cisplatin regarding acute and late toxicities in terms of oral mucositis grade ≥ 2 (*p* = 0.34) or grade ≥ 3 (*p* = 0.38), dermatitis grade ≥ 2 (*p* = 0.27) or grade ≥ 3 (*p* = 1.00), nausea grade ≥ 2 (*p* = 0.50) or grade ≥ 3 (*p* = 0.27), xerostomia grade ≥ 2 (*p* = 0.74) or grade ≥ 3 (*p* = 1.00), lymphedema grade ≥ 2 (*p* = 1.00) or grade ≥ 3 (*p* = 1.00), and hematotoxicity grade ≥ 2 (*p* = 0.74), grade ≥ 3 (*p* = 0.74) or grade 4 (*p* = 0.60) (Table 8).

In the subgroup analysis of the 79 patients receiving definitive chemoradiation, the LRC rates after 1, 2, and 3 years were 71%, 55%, and 49% in the carboplatin group (*n* = 23) vs. 70%, 61%, and 58% in the cisplatin group (*n* = 56) (*p* = 0.53). The MFS rates were 85%, 75%, and 69% in the carboplatin group vs. 94%, 87%, and 84% in the cisplatin group (*p* = 0.058), and the OS rates were 91%, 81%, and 75% vs. 87%, 80%, and 74% (*p* = 0.78). In the subgroup analysis of the 97 patients receiving adjuvant chemoradiation, the LRC rates after 1, 2, and 3 years were 85%, 85%, and 85% in the carboplatin group (*n* = 22) vs. 92%, 90% and 90% in the cisplatin group (*n* = 75) (*p* = 0.54). The MFS rates were 90%, 82%, and 82% in the carboplatin group vs. 86%, 80%, and 75% in the cisplatin group (*p* = 0.63), and the OS rates were 86%, 86%, and 77% in the carboplatin group vs. 88%, 84%, and 82% in the cisplatin group (*p* = 0.84).

## 4. Discussion

Standard chemoradiation for SCCHN includes cisplatin, commonly consisting of 100 mg/m^2^ on one day, administered every 3 weeks [1,2,3]. Several studies suggested that the minimum cumulative cisplatin dose during a course of radiotherapy should be 200 mg/m^2^ [43,44,45]. Therefore, other cisplatin regimens including weekly doses of 30–40 mg/m^2^ or two courses of fractionated cisplatin, as used in the present study, are also relatively common. However, cisplatin can be associated with significant morbidity, particularly nephrotoxicity. Therefore, many patients are not suitable for this agent. Since the addition of concurrent chemotherapy to radiotherapy improves the outcomes of treatment in patients with SCCHN, the replacement of cisplatin by other systemic agents is considered a better option than radiotherapy alone, irrespective of the radiation regimen [1,2,3,9,11,12]. Several options of systemic therapies without cisplatin exist, for example, carboplatin plus/minus 5-FU or paclitaxel, mitomycin C plus 5-FU, or cetuximab. According to previous studies, 5-FU-based regimens were quite toxic, and the anti-epidermal-growth-factor-receptor antibody cetuximab appeared less effective than “classic” chemotherapies [4,5,6,7,8,9,10,11,12,13,18,19,20,21,22,23,24,25,26,27,28,29]. Thus, carboplatin plus paclitaxel and carboplatin alone remain of the options mentioned above. In the study of Beckham et al., which compared different platin-based chemotherapy regimens and cetuximab in 316 patients with HPV-negative head-and-neck cancer, carboplatin alone was not inferior to carboplatin plus 5-FU or paclitaxel with respect to LRC (HR: 0.69, 95% CI: 0.23–2.08, *p* = 0.51), MFS (HR: 1.47, 95% CI: 0.32–6.68, *p* = 0.62), and OS (HR: 1.30, 95% CI: 0.56–3.04, *p* = 0.54) [23]. Whether carboplatin alone was associated with less toxicity than the combined regimen, was not stated but may be suspected. Thus, chemoradiation with carboplatin may be a reasonable alternative for patients not suitable for cisplatin.

Several studies have compared carboplatin alone and cisplatin alone for the chemoradiation of head-and-neck cancer [30,31,32,33,34,35,36,37,38,39,40,41]. Some of these studies were additionally included in one or two meta-analyses from 2016 [46,47]. However, the results of the studies and meta-analyses were conflicting. Of the studies published prior to 2015, three randomized trials and one matched-pair study found that carboplatin was not inferior to cisplatin [30,31,34,36]. Moreover, in a randomized phase II trial from 2004 including 119 patients with SCCHN, weekly carboplatin resulted in a significantly better 5-year LRC (56.2% vs. 35.5%) and a non-significantly better OS (71.4% vs. 66.0%) when compared to daily low-dose cisplatin [33]. Only two studies published before 2015 suggested that carboplatin was less effective than cisplatin [32,35]. One study was retrospective in nature and limited to cancer of the oropharynx or oral cavity [35]. Of the 215 patients screened, only 106 patients (49%) had complete data and were included in the analyses. This may have led to a selection bias. Moreover, non-significantly more patients in the carboplatin group had cancer of the oral cavity, which generally has a worse prognosis than cancer of the oropharynx. In addition, important prognostic factors including HPV status, smoking before and during the radiotherapy course, and the pre-treatment hemoglobin level were not considered. Therefore, the results of this retrospective study should be interpreted with caution [35]. The other study had a higher quality, being a randomized phase III trial [32]. It compared radiotherapy alone (41 patients) to chemoradiation with 100 mg/m^2^ of cisplatin given on days 2, 22, and 42 (45 patients) and chemoradiation with carboplatin (AUC 7) given on days 2, 22, and 42 (38 patients). In that trial, carboplatin-based chemoradiation was superior to radiotherapy alone but inferior to cisplatin-based treatment with respect to time to progression and median OS [32]. The two meta-analyses mentioned above were also limited to studies published before 2015 [46,47]. Both meta-analyses found that LRC and OS were not significantly different after chemoradiation with carboplatin or cisplatin. However, in a subgroup analysis of non-nasopharynx cancer performed in one meta-analysis, cisplatin resulted in significantly better 3-year OS (HR: 0.66; 95% CI: 0.48–0.91; *p* = 0.01) [46].

Since radiotherapy of SCCHN has undergone technical improvements during the last 10 years, including the increasing use of high-precision radiotherapy with VMAT (as in the present study), it appears reasonable to take a separate look at studies published more recently. No randomized trials but several retrospective studies were identified that were published between 2017 and 2023 [37,38,39,40,41]. Three of these studies found that chemoradiation with carboplatin was similarly effective as cisplatin-based treatment [37,38,41]. One study suggested similar efficacy for stage I or II disease, but a superiority of cisplatin for stage III disease [39]. In another study that compared chemoradiation with high-dose cisplatin (cumulative dose of ≥200 mg/m^2^), chemoradiation with low-dose cisplatin (<200 mg/m^2^), chemotherapy with carboplatin, and radiotherapy alone, high-dose cisplatin-based treatment was associated with significantly better OS than the other three regimens [40]. However, the patients in the carboplatin group and the radiotherapy alone group were significantly older and had a higher comorbidity index than patients of the high-dose cisplatin group, which likely led to a bias in favor of high-dose cisplatin [40].

Considering the conflicting results regarding the potential role of chemoradiation with carboplatin alone, it becomes obvious that additional studies are required to properly define its value. Therefore, we performed the present study. According to its results, concurrent chemoradiation with carboplatin was not inferior to concurrent cisplatin with respect to the toxicities and treatment outcomes in terms of LRC, MFS, and OS. In contrast to the chemotherapy regimen, lower primary tumor stage, lower nodal stage, and HPV positivity were independently associated with improved treatment outcomes on multivariable analyses. A trend was found for an association between higher KPS and better OS. Such associations were already found in previous studies, which demonstrates the consistency of the data of our present study [14,42,43,48,49].

When interpreting the results of the previous studies, one should be aware that most of those studies comparing carboplatin and cisplatin for chemoradiation of SCCHN were performed in patients receiving definitive treatment. Only two comparative studies also included patients who received chemoradiation in an adjuvant situation [33,36]. One of these studies (a phase II trial) found that carboplatin was superior to cisplatin, and the other study (retrospective matched-pair analysis) suggested that both agents were similarly effective. In addition, a non-comparative study that also included patients in an adjuvant situation found that chemoradiation with carboplatin was well tolerated [27]. Moreover, a small retrospective study of 47 patients suggested that chemoradiation with carboplatin resulted in non-significantly better median progression-free survival (43 vs. 12 months) and OS (92 vs. 36 months) than radiotherapy combined with cetuximab [29]. However, the small sample size and the retrospective design must be considered when interpreting these results. In contrast to these studies supporting the use of carboplatin, adjuvant chemoradiation with weekly carboplatin appeared not significantly superior to radiotherapy alone with respect to disease-free survival (DFS) at 2 and 5 years in a phase III trial [50]. The 2-year DFS rates were 71% with and 58% without carboplatin (*p* = 0.27), and 5-year DFS rates were 53% and 49% (*p* = 0.72). The 2-year OS rates were 74% vs. 51% (*p* = 0.04), and 5-year OS rates were 47% vs. 41% (*p* = 0.61), respectively. However, the trial was prematurely closed due to slow accrual after 76 of the planned 200 patients. Moreover, the patients were treated in the pre-VMAT era, namely between 1992 and 2002. Therefore, the results of this phase III trial may be of limited validity, which is also stated by the authors. Considering the lack of data in an adjuvant situation, we performed subgroup analyses in patients receiving definitive chemoradiation and patients receiving adjuvant treatment. According to these analyses, a trend was found for improved MFS with cisplatin in patients receiving definitive chemoradiation. However, carboplatin was not significantly inferior to cisplatin in both subgroups with respect to LRC, MFS, or OS. Thus, it may be an option for patients unable to receive cisplatin, irrespective of the upfront surgery.

### Limitations of the Study

When interpreting the results of our study, its limitations should be regarded. Limitations include the use of a cisplatin regimen (two courses of cisplatin, consisting of 20 mg/m^2^/d1–5 or 25 mg/m^2^/d1–4), which is not the standard in many centers worldwide. Since all patients were treated with VMAT, a modern precision radiotherapy technique, it remains unclear whether the results of this study can be generalized to patients treated with other less modern but still widely used radiotherapy techniques such as 3D-conformal radiotherapy or intensity-modulated radiotherapy. Moreover, the sample size in the carboplatin group was comparably small. Another limitation of our study is the difference between both the treatment groups regarding the length of the follow-up period, which may have led to a bias. In addition, the retrospective study design may have introduced (hidden) selection biases. In our present study, all but two baseline characteristics were balanced between the treatment groups. The distributions of age and histologic grading were more favorable in the cisplatin group, which supports the findings that carboplatin may be not inferior. Moreover, most patients in the carboplatin group had a decreased pre-treatment renal function. Despite the results in favor of carboplatin, its real value can only be properly identified in a prospective randomized trial using modern radiotherapy techniques, preferably VMAT.

## 5. Conclusions

Although patients who received carboplatin had worse pre-treatment renal function, were older, and had more aggressive (less differentiated) tumors than patients treated with cisplatin, carboplatin did not result in significantly worse treatment outcomes (LRC, LFS, OS) or toxicities. Given the limitations of this study, concurrent chemoradiation with carboplatin alone appears an option for patients with SCCHN not suitable for cisplatin-based treatment.

## Figures and Tables

**Table 1:** Distributions of pre-treatment patient and tumor characteristics in the carboplatin group (*n* = 45) and the cisplatin group (*n* = 131).

Characteristic	Carboplatin *n* Patients (%)	Cisplatin *n* Patients (%)	*p*-Value
Age			0.007
≤62 years	16 (36)	77 (59)	
≥63 years	29 (64)	54 (41)	
Gender			0.15
Female	12 (27)	22 (17)	
Male	33 (73)	109 (83)	
Karnofsky performance status			0.50
≤80	18 (40)	60 (46)	
90–100	27 (60)	71 (54)	
Tumor site			0.40
Oropharynx/oral cavity	26 (58)	88 (67)	
Hypopharynx/larynx	13 (29)	33 (25)	
Both	6 (13)	10 (8)	
Primary tumor stage			0.95
T1–2	12 (27)	38 (29)	
T3	12 (27)	33 (25)	
T4	21 (47)	60 (46)	
Nodal stage			0.80
N0-2a	15 (33)	41 (31)	
N2b-3	30 (67)	90 (69)	
Histologic grade ^a^			0.017
G1–2	17 (39)	75 (60)	
G3	27 (61)	51 (40)	
HPV status ^b^			0.23
Negative	24 (71)	53 (59)	
Positive	10 (29)	37 (41)	
Upfront surgery			0.33
No	23 (51)	56 (43)	
Yes	22 (49)	75 (57)	
Pre-treatment history of smoking ^c^			0.69
No	8 (18)	18 (15)	
Yes	37 (82)	100 (85)	
Smoking during chemoradiation ^d^			0.18
No	32 (71)	71 (60)	
Yes	13 (29)	48 (40)	
Pre-treatment hemoglobin level			0.18
<12 g/dL	23 (51)	52 (40)	
≥12 g/dL	22 (49)	79 (60)	

HPV: human papilloma virus. The *p*-values were obtained with the Chi-square test. ^a^ Unknown in 6 patients; ^b^ unknown in 52 patients; ^c^ unknown in 13 patients; ^d^ unknown in 12 patients.

**Table 2:** Loco-regional control rates related to the type of chemotherapy and other characteristics (univariable analyses).

Characteristic	Loco-Regional Control Rates (%)			*p*-Value
	At 1 Year	At 2 Years	At 3 Years	
Type of chemotherapy				0.21
Carboplatin	78	69	65	
Cisplatin	83	77	76	
Age				0.90
≤62 years	84	72	72	
≥63 years	79	79	74	
Gender				0.14
Female	97	94	81	
Male	78	71	71	
Karnofsky performance status				0.049
≤80	78	69	69	
90–100	84	80	76	
Tumor site				0.19
Oropharynx/oral cavity	85	80	78	
Hypopharynx/larynx	73	65	65	
Both	79	70	56	
Primary tumor stage				<0.001
T1–2	96	93	93	
T3	87	84	79	
T4	69	59	57	
Nodal stage				0.004
N0-2a	94	94	90	
N2b-3	75	66	65	
Histologic grade ^a^				0.77
G1–2	79	73	68	
G3	83	76	76	
HPV status ^b^				0.001
Negative	80	70	67	
Positive	96	93	93	
Upfront surgery				<0.001
No	71	59	55	
Yes	90	89	89	
Pre-treatment history of smoking ^c^				0.64
No	88	83	77	
Yes	81	75	73	
Smoking during chemoradiation ^d^				0.26
No	84	79	76	
Yes	77	69	69	
Pre-treatment hemoglobin level				0.40
<12 g/dL	80	75	68	
≥12 g/dL	83	75	75	
Completion of chemotherapy				0.078
No	73	65	61	
Yes	85	79	78	

HPV: human papilloma virus. The *p*-values were calculated with the log-rank test. ^a^ Unknown in 6 patients; ^b^ unknown in 52 patients; ^c^ unknown in 13 patients; ^d^ unknown in 12 patients.

**Table 3:** Multivariable analysis of loco-regional control.

Characteristic	Hazard Ratio	95% Confidence Interval	*p*-Value
Karnofsky performance status	1.03	0.45–2.38	0.94
Primary tumor stage	2.10	1.07–4.13	0.031
Nodal stage	2.40	0.80–7.18	0.12
HPV status	3.46	1.02–11.76	0.047
Upfront surgery	1.95	0.65–5.85	0.23

HPV: human papilloma virus.

**Table 4:** Metastases-free survival rates related to type of chemotherapy and other characteristics (univariable analyses).

Characteristic	Metastases-Free Survival Rates (%)			*p*-Value
	At 1 Year	At 2 Years	At 3 Years	
Type of chemotherapy				0.34
Carboplatin	87	78	74	
Cisplatin	89	83	78	
Age				0.48
≤62 years	89	82	80	
≥63 years	88	81	74	
Gender				0.45
Female	97	89	79	
Male	87	80	77	
Karnofsky performance status				0.031
≤80	84	74	69	
90–100	93	87	84	
Tumor site				0.88
Oropharynx/oral cavity	93	82	78	
Hypopharynx/larynx	83	83	73	
Both	80	80	80	
Primary tumor stage				0.48
T1–2	92	87	83	
T3	90	79	79	
T4	86	79	72	
Nodal stage				0.013
N0-2a	92	92	92	
N2b-3	87	77	71	
Histologic grade ^a^				0.26
G1–2	88	84	82	
G3	89	78	71	
HPV status ^b^				0.008
Negative	88	73	70	
Positive	95	95	95	
Upfront surgery				0.84
No	92	83	79	
Yes	87	81	76	
Pre-treatment history of smoking ^c^				0.29
No	92	92	92	
Yes	88	78	75	
Smoking during chemoradiation ^d^				0.13
No	89	84	82	
Yes	86	73	69	
Pre-treatment hemoglobin level				0.32
<12 g/dL	88	82	73	
≥12 g/dL	90	82	80	
Completion of chemotherapy				0.84
No	87	79	79	
Yes	90	83	77	

HPV: human papilloma virus. The *p*-values were calculated with the log-rank test. ^a^ Unknown in 6 patients; ^b^ unknown in 52 patients; ^c^ unknown in 13 patients; ^d^ unknown in 12 patients.

**Table 5:** Multivariable analysis of metastases-free survival.

Characteristic	Hazard Ratio	95% Confidence Interval	*p*-Value
Karnofsky performance status	1.19	0.47–3.00	0.71
Nodal stage	10.20	1.35–76.97	0.024
HPV status	3.97	1.14–13.89	0.030

**Table 6:** Overall survival rates related to type of chemotherapy and other characteristics (univariable analyses).

Characteristic	Overall Survival Rates (%)			*p*-Value
	At 1 Year	At 2 Years	At 3 Years	
Type of chemotherapy				0.64
Carboplatin	89	83	75	
Cisplatin	88	83	79	
Age				0.093
≤62 years	91	86	83	
≥63 years	84	78	73	
Gender				0.59
Female	91	91	82	
Male	87	81	77	
Karnofsky performance status				0.003
≤80	79	72	67	
90–100	95	91	86	
Tumor site				0.47
Oropharynx/oral cavity	88	85	82	
Hypopharynx/larynx	87	78	74	
Both	88	80	64	
Primary tumor stage				0.14
T1–2	89	89	89	
T3	89	82	77	
T4	86	78	70	
Nodal stage				0.92
N0-2a	86	86	80	
N2b-3	89	81	76	
Histologic grade ^a^				0.49
G1–2	89	82	76	
G3	87	84	79	
HPV status ^b^				0.021
Negative	80	75	70	
Positive	96	93	87	
Upfront surgery				0.31
No	88	80	74	
Yes	87	85	81	
Pre-treatment history of smoking ^c^				0.33
No	88	88	88	
Yes	87	80	74	
Smoking during chemoradiation ^d^				0.009
No	91	86	84	
Yes	81	73	62	
Pre-treatment hemoglobin level				0.093
<12 g/dL	81	77	75	
≥12 g/dL	93	87	80	
Completion of chemotherapy				0.060
No	81	73	73	
Yes	91	87	80	

HPV: human papilloma virus. The *p*-values were calculated with the log-rank test. ^a^ Unknown in 6 patients; ^b^ unknown in 52 patients; ^c^ unknown in 13 patients; ^d^ unknown in 12 patients.

**Table 7:** Multivariable analysis of overall survival.

Characteristic	Hazard Ratio	95% Confidence Interval	*p*-Value
Karnofsky performance status	1.79	0.80–3.97	0.15
HPV status	1.96	0.73–5.26	0.18
Smoking during chemoradiation	1.68	0.73–3.88	0.23

HPV: human papilloma virus.

**Table 8:** Comparison of carboplatin and cisplatin with respect to acute and late toxicities.

Toxicity	Carboplatin *n* Patients (%)	Cisplatin *n* Patients (%)	*p*-Value
Oral mucositis ^a^			
Grade ≥ 2	25 (64)	88 (72)	0.34
Grade ≥ 3	12 (31)	29 (24)	0.38
Dermatitis ^b^			
Grade ≥ 2	32 (76)	103 (84)	0.27
Grade ≥ 3	14 (33)	41 (33)	1.00
Xerostomia ^c^			
Grade ≥ 2	13 (30)	35 (28)	0.74
Grade ≥ 3	2 (5)	6 (5)	1.00 *
Cervical lymph edema ^d^			
Grade ≥ 2	4 (10)	12 (10)	1.00 *
Grade ≥ 3	0 (0)	1 (1)	1.00 *
Nausea ^e^			
Grade ≥ 2	6 (14)	13 (10)	0.50
Grade ≥ 3	2 (5)	2 (2)	0.27 *
Hematotoxicity ^f^			
Grade ≥ 2	33 (73)	92 (71)	0.74
Grade ≥ 3	11 (24)	35 (27)	0.74
Grade 4	2 (4)	3 (2)	0.60 *

^a^ Unknown in 15 patients; ^b^ unknown in 11 patients; ^c^ unknown in 6 patients; ^d^ unknown in 8 patients; ^e^ unknown in 6 patients; ^f^ unknown in 1 patient; * calculated with Fisher’s exact test.

## Data Availability

The data cannot be shared due to data protection regulations. Only evaluation of anonymized data is allowed according to the responsible ethics committee.
